# Multimodal Prediction of Future Depressive Symptoms in Adolescents

**DOI:** 10.21203/rs.3.rs-6585192/v1

**Published:** 2025-10-31

**Authors:** Linshanshan Wang, Ningxuan Zhou, Nigel M. Jaffe, Kristina Pidvirny, Anna O. Tierney, Hadar B. Fisher, Francesca Morfini, Erika E. Forbes, Diego A. Pizzagalli, Tianxi Cai, Christian A. Webb

**Affiliations:** Harvard T.H. Chan School of Public Health; Harvard T.H. Chan School of Public Health; McLean Hospital; McLean Hospital; McLean Hospital; McLean Hospital; McLean Hospital; University of Pittsburgh; McLean Hospital; Harvard T.H. Chan School of Public Health; McLean Hospital

## Abstract

**Background:**

Depression rates surge during adolescence. Early identification of youth at increased risk for depression is crucial for timely intervention and, ideally, prevention. This study aims to improve the prediction of future depressive symptoms in adolescents by using a multimodal approach that integrates relevant clinical, demographic, behavioral, and neural characteristics.

**Methods:**

103 adolescents (ages 12–18; 72.8% female) underwent a baseline assessment including self-report questionnaires, ecological momentary assessment, a clinical interview, and behavioral and neural measures of reward responsiveness. We used nested cross-validation to compare machine learning approaches as well as conventional linear regression in predicting depressive symptoms (Center for Epidemiological Studies Depression Scale [CES-D] and the Mood and Feelings Questionnaire [MFQ]) at a 3-month follow-up.

**Results:**

For the prediction of CES-D depression scores, the best performing model was a multivariable linear regression using as predictors five principal component scores from a principal component analysis of baseline variables (RMSE = 6.501, R^2^ = 0.688). For the MFQ, the best performing model was a univariable linear regression with baseline MFQ scores as the sole predictor (RMSE = 8.054, R^2^ = 0.671). A factor analysis revealed that items assessing melancholic features were most predictive of future depressive symptoms.

**Conclusion:**

More complex machine learning approaches did not outperform regression in predicting future depression. The integration of relevant multimodal predictors reveals which adolescent characteristics (e.g., melancholic features and physical anxiety) have a larger contribution to predicting short-term future depression. Future studies are needed with larger sample sizes and longer follow-up periods to provide a more comprehensive test of such models.

## Introduction

Rates of major depressive disorder (MDD) among adolescents and young adults have risen substantially over the last 15 years [1]. More recently, evidence points to further increases in child and adolescent depression symptoms during the COVID pandemic [2]. This notable increase underscores the critical need for early identification of adolescents at risk for depression, which could inform early intervention and preventative efforts.

Despite widespread awareness of a mental health epidemic afflicting youth, researchers have not yet succeeded in developing statistical models that can reliably forecast depressive symptoms in adolescents. Comparable systems already exist for certain physical health problems. For example, the Predicting Risk of Cardiovascular Disease Events (PREVENT) calculator is an algorithm incorporating several relevant predictors (e.g., age, sex, cholesterol, current smoking, and body mass index) as inputs to provide personalized estimates of a patient’s risk of developing cardiovascular disease outcomes such as coronary heart disease or heart failure [3]. A similar predictive model for depressive symptoms would represent an important step forward for the field, as more reliable identification of early warning signs of future depression (i.e., “red flags”) may mitigate MDD’s negative consequences by allowing the timely delivery of preventive interventions. In addition, a fuller understanding of the extent to which certain factors (e.g., demographic and clinical characteristics, environmental/social variables or neural features) each contribute to depression risk might allow for the development of algorithms that offer personalized prediction of future symptoms, with the potential for tailoring preventative interventions to an individual’s particular risk profile (e.g., intervention recommendations may differ if neuroticism vs. rumination vs. anhedonia were especially elevated in a given individual) [4].

In an effort to better understand and predict the onset of depression during adolescence, previous studies have identified a range of risk factors that prospectively predict depression (for a recent review see [5]). Studies have typically only examined one or a few risk factors in isolation. For instance, previous studies have identified a range of predictors of depression, including demographic variables such as age and gender [6, 7], clinical characteristics like depressive symptoms, anhedonia, anxiety symptoms, and rumination [8–11], socioeconomic status [12], pubertal status [13], as well as neural markers such as decreased striatal activation to reward [14, 15]. Considering each variable in isolation may account for only limited variance in future depression outcomes. Since depression is a heterogeneous disorder emerging from a complex interplay of environmental/social, psychological, and biological factors, it is difficult to predict the onset of depression based on any single variable [16]. Therefore, there is a need for a more comprehensive, data-driven approach that considers multiple risk markers simultaneously to improve predictive accuracy and to better reflect the reality of depression’s multifaceted etiology.

Recently, machine learning techniques have gained traction in predicting depression. These approaches are well-suited to handling a large number of predictor variables (including multicollinearity among variables) and modeling complex relations such as interactions and other non-linear associations that traditional statistical methods might overlook (e.g., in ordinary least squares (OLS) regression if relevant interactions and non-linear relations are not explicitly specified). Prior studies using machine learning have shown promising results. For instance, a recent large study achieved area under the receiver operating characteristic curve (AUROC) values of 0.72–0.74 in identifying depression in 6,310 children and adolescents aged 4–17 [17]. However, while these studies incorporated several predictors, they typically collected data within one modality (e.g., all self-reports) anddid not combine multi-modal data from several relevant sources [18], for an exception see [19]. An important next step involves determining if combining measures of depression-relevant characteristics assessed from various modalities – such as conventional self-report questionnaires (e.g., assessing clinical, demographic, and personality characteristics), ecological momentary assessments (EMAs; e.g., affect dynamics in daily life), behavioral tasks (e.g., reward learning and cognitive control abilities), and neuroimaging (e.g., reward-related brain activation) – can enhance our ability to predict depression in adolescents.

In addition, most recent studies employing machine learning to examine multiple predictors of depression tested associations with *current* depression. Longitudinal studies are required to study the predictive value of these factors for the onset of future depressive symptoms. One exception is a recent study that used machine learning to combine various modalities such as clinical data, cognitive assessments, environmental factors, and structural magnetic resonance imaging (MRI) to predict depression onset at a 2-year and 5-year follow-up [19]. The study used penalized logistic regression and found that baseline depressive symptoms, female sex, neuroticism, stressful life events, and the surface area of the supramarginal gyrus were the strongest predictors. The predictive model achieved an AUROC of 0.68–0.72. However, penalized logistic regression, while effective in handling multicollinearity and overfitting, does not account for complex interactions and nonlinear associations (unless they are explicitly modeled). Other algorithms, such as decision-tree based models (e.g., random forest) are well-designed to do so and may yield better predictive performance, highlighting the value of comparing a range of machine learning approaches.

The current study aims to address the abovementioned limitations by combining multimodal data, including depression-relevant self-reports/EMA (e.g., assessing neuroticism, rumination, stress, and affect dynamics), behavioral assessments (reward learning), and neural measures (neural reward sensitivity), to prospectively predict future depressive symptoms in youth. The integration of different data sources may also help in identifying novel predictors and interactions that may be important for early detection and intervention. In addition, the current study will compare various machine learning approaches, as well as conventional regression, to identify which best predicts depressive symptoms in adolescents.

## Methods and Materials

### Participants

Participants were 103 English-speaking adolescents aged 12–18 (75 female, 28 male; *m*_*age*_ = 16.0, *SD* = 1.9) recruited from the greater Boston area for two larger studies that recruited typically developing (non-anhedonic) adolescents (n = 68 included in the current study), as well as adolescents with elevated levels of anhedonia (n = 35 included in the current study). Exclusion criteria included history or current diagnosis of any of the following DSM-5 psychiatric illnesses: major depressive disorder, schizophrenia spectrum or other psychotic disorder, bipolar disorder, substance or alcohol use disorder within the past 12 months or lifetime severe substance or alcohol use disorder, and current diagnosis of anorexia nervosa or bulimia nervosa. Psychotropic medications were exclusionary, with the exception of stable-dose (at least one month) selective serotonin reuptake inhibitors (SSRIs) (n = 2; see Table 1). See Supplement for additional exclusion criteria.

### Procedure

All procedures were approved by the Mass General Brigham IRB. Written informed consent was provided by participants who were 18 years of age, as well as from parents of participants who were under 18 years of age, along with the participant’s written informed assent. The authors assert that all procedures contributing to this work comply with the ethical standards of the relevant national and institutional committees on human experimentation and with the Helsinki Declaration of 1975, as revised in 2008. At the baseline session, either in-person or over Zoom, participants were administered a semi-structured clinical interview, the Kiddie Schedule for Affective Disorders and Schizophrenia (K-SADS; [20]), and completed self-report measures. Following the baseline session, participants completed an MRI scan session including a Reward Task [21] functional MRI (fMRI) probing neural response to the anticipation and receipt of, loss of, or no change in monetary reward vs. losses. After the MRI session, participants performed a Probabilistic Reward Task (PRT; [22]) assessing reward learning. See Supplement for further details on MRI acquisition, task design, and MRI data processing. At the MRI scan session, participants installed the MetricWire App on their smartphones to complete EMAs asking about current positive affect (PA) and negative affect (NA). As described in Murray et al. [23], following the fMRI scan session EMA surveys were delivered 2–3 times per day whereby participants were randomly signaled during two timeslots (4pm to 6:30pm and 6:30pm to 9pm) during a 5-day period (Thursday - Monday). A third survey was sent on weekends (11am-4pm). Three months after the baseline session, participants completed the baseline self-report measures again, including depression measures (see below).

#### Outcome Measures:

Two self-report depression outcome measures were assessed at the 3-month follow-up: Center for Epidemiological Studies Depression Scale (CES-D; [24]) total score, assessing past-week symptoms, and the Mood and Feelings Questionnaire – Child Self-Report – Long Version (MFQ; [25]) total score, assessing symptoms over the past two weeks. Both measures are widely used but differ on several dimensions, including content (e.g., the MFQ has greater coverage of DSM-5 symptoms), sensitivity (the CESD has been shown to be relatively sensitive in detecting depressive symptoms at the lower severity range), and timeframe (assessing symptoms over the past week vs. past 2 weeks) [26–28].

### Baseline Predictor Variables

#### Clinical self-report scales

Depression (CES-D and MFQ), anhedonia (Snaith-Hamilton Pleasure Scale [SHAPS; [29]] total score), rumination (Children’s Response Styles Questionnaire [CRSQ; [30]] rumination subscale score), perceived stress (Perceived Stress Scale [PSS; [31]] total score), physical anxiety (Multidimensional Anxiety Scale for Children [MASC; [32]] subscale score), social anxiety (MASC social subscore), separation anxiety (MASC separation subscore), harm avoidance (MASC harm subscore), negative life events (Adolescent Life Event Questionnaire-Revised [ALEQ-R; [33]] total score), extraversion (NEO Five-Factor Inventory-3 [NEO-FFI-3; [34]] extraversion factor), and neuroticism (NEO-FFI-3 neuroticism factor).

##### Ecological Momentary Assessment:

Participants rated emotions on a 5-point Likert scale, with mean PA and NA calculated from respective emotions. See Supplement for full details. Several affect dynamic measures previously linked to depression were also included: the variability in PA and NA were measured using the standard deviation (SD) and mean square successive difference (MSSD; [35–38]), and we also included temporal dependency (autocorrelation; [39]) of PA and NA as a measure of emotional inertia.

#### Clinical Interview

Participants were administered the K-SADS. As noted above, we included participants from a no anhedonia vs. elevated anhedonia group (binary variable). Elevated anhedonia was defined as having a K-SADS anhedonia item (from the MDD module) score greater than one.

#### Neuroimaging

Participants completed an fMRI monetary reward task assessing neural responses during reward anticipation and outcome [21, 23, 40]. See Supplement for task details.

##### Behavioral:

A reward learning task (Probabilistic Reward Task [PRT]) previously validated in adolescents [41–43] was used. We included two variables as predictors: reward learning (change in response bias over the course of the task computed as Response Bias in block 2 minus Response Bias in block 1) and mean response bias (averaging the response bias score over the two blocks). For task details, see [40].

#### Demographic

Self-reported age and sex.

#### Pubertal

Pubertal status (Tanner Staging Questionnaire [TSQ; [44]] total score).

### Statistical Analysis

#### Concurrent correlations with depressive symptoms at baseline

**Figure 1:**
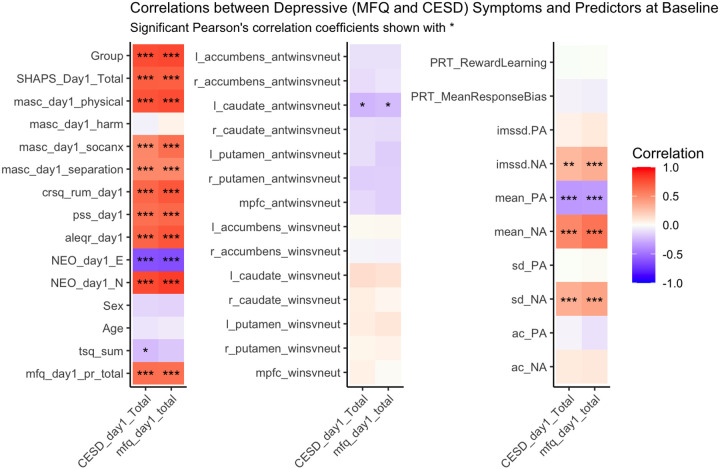
Concurrent correlation between depressive symptoms and other predictors at baseline. Significance level is indicated by *. * <0.05, ** <0.01, ***<0.001

Prior to testing the relation between baseline predictors and future (3 month) depressive (CESD and MFQ) symptoms, we first examined the concurrent correlations between depression scores and other predictor variables at baseline (see Fig. 1 for correlation coefficients and *p*-values).

#### Predicting future (3 months) depressive symptoms

**Figure 2:**
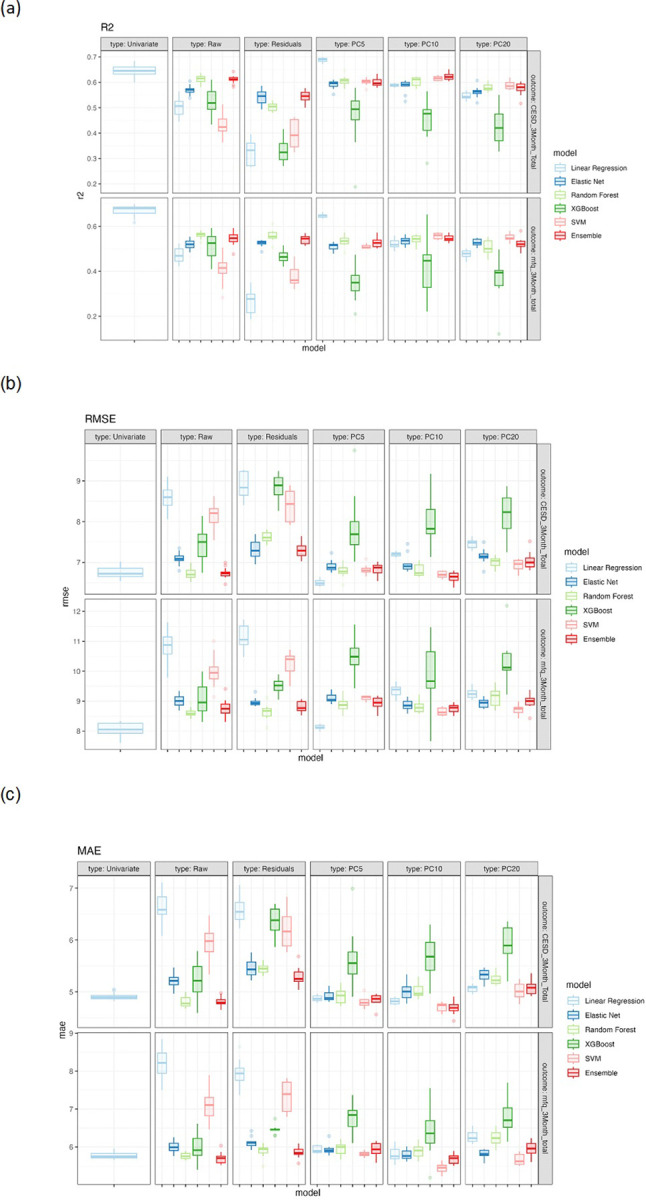
Schematic of data processing and modeling pipeline.

See Fig. 2 for a schematic of the data processing and modeling pipeline.

##### Data Preprocessing

We standardized all variables to have means of 0 and standard deviations of 1 (Data type: *Raw*). To reduce the potential instability in model training due to the high correlations between the baseline predictors, we re-ran analyses applying two separate approaches to data pre-processing: (1) To remove the confounding influence of baseline depression severity from our other predictors, for each variable other than the baseline depression scores (MFQ or CESD), we regressed the variable against baseline depression and took the residuals as the feature value. We trained the models using the residualized features along with baseline depression (Data type: *Residuals*). (2) To reduce the number of predictors, we applied principal component analysis (PCA) to all baseline predictors (across all modalities), then evaluated the first 5, 10, or 20 principal component scores as features (Data type: *PC5, PC10, PC20*). See “data type” column in Table 2. See **Supplementary Fig. 3** for the cumulative variance explained by the top principal components.

##### Model Training and Validation

**Figure 3:**
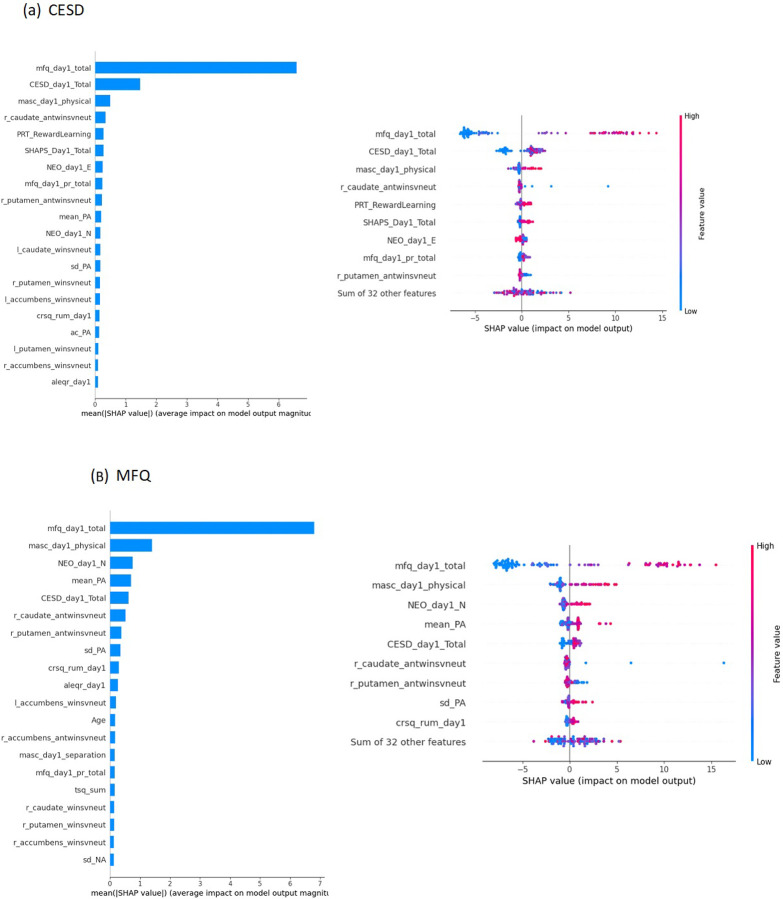
Performance of models for predicting depressive symptoms at 3 months follow-up, as measured by (a) R2: R squared, (b) RMSE: root mean squared error, and (c) MAE: mean absolute error. Results are summarized over 30 repetitions. Univariate: model with respective baseline depression measures as the sole predictor, Raw: all normalized baseline variables as predictors, Residuals: residualized baseline variables as predictors, PC5: first 5 principal component scores, PC10: first 10 principal component scores, PC20: first 20 principal component scores.

We fit five different predictive models with all baseline predictors: (1) conventional linear regression, (2) linear regression with elastic net penalty [45], (3) random forest [46], (4) XGBoost [47], and (5) Support vector machine (SVM, [48]). We further trained an ensemble learning model [49] which combines predictions from the base models (linear regression, elastic net, random forest, XGBoost and SVM) in an effort to maximize predictive performance. We also compared these models to a univariable model with baseline depression (CESD or MFQ) total score as the only predictor. We fit a linear regression to predict the depression outcome at 3 months with the corresponding baseline measure as the sole predictor variable. To perform model training with hyperparameter optimization while robustly evaluating the model performance, we performed *nested* cross-validation (CV; see Supplement for details). Preprocessing steps (standardization, residualization, and PCA) were performed within the training folds during nested cross-validation. This ensures that no information from the test folds was used to inform any transformation. See Table 2 (also see Fig. 3) for results.

#### Feature Importance

To assess the most influential features in the multivariable model for the prediction of depression, SHapley Additive exPlanations (SHAP) were implemented, and the top ten most influential features were reported for the random forest model, which achieved the highest performance using standardized predictors (data type: *Raw*). See Supplement for additional details.

#### Factor Analysis

We conducted two exploratory factor analyses (EFA) to examine factors underlying the depression questionnaire (MFQ and CESD) items at baseline, in an effort to determine which depression features, if any, were most predictive of future depressive symptoms. See Supplement for details.

## Results

### Participant characteristics

Participants were predominantly White (*n* = 64, 62.1%), non-Hispanic (*n* = 92, 89.3%) teens, most of whom were assigned female at birth (*n* = 75, 72.8%). Baseline anhedonia (SHAPS: *M* = 23.0, *SD* = 7.4) and depression symptoms (CES-D: *M* = 12.3, *SD* = 11.1) were relatively mild in severity. We report additional demographic and clinical characteristics in Table 1. At the 3-month timepoint, CES-D scores were slightly higher (*M* = 13.3, *SD* = 13.1) than at the baseline assessment, but below the conventional cutoff score of 16 [24], with substantial variability at both timepoints.

### Baseline correlations

We report Spearman correlation coefficient between depression symptoms and other predictive variables at baseline, along with the p-values in Fig. 1. Both depression measures (CESD and MFQ) exhibit statistically significant correlations with a range of self-report measures: anhedonia (SHAPS), anxiety facets (MASC physical, social, and separation anxiety subscores), rumination (CRSQ) perceived stress (PSS), negative life events (ALEQR), and neuroticism (NEO), ranging from *r = 0.4 to 0.75*. Strong negative correlations were found between both baseline depression measures and extraversion (NEO) (CESD: r = −0.58, p = 2e-10; MFQ: r = −0.58, p = 1e-10). Among the EMA measures, lower mean PA and higher mean *and* variability in NA (both SD and MSSD) were significantly associated with higher depression. Among the neuroimaging variables, only blunted striatal (left caudate) response to the anticipation of rewards was significantly correlated with greater depressive symptoms on both measures. Our behavioral measure of reward learning (PRT) was not correlated with depression (nor anhedonia).

### Principal Component Analysis

The PCA decomposition (see **Supplementary Fig. 3**) showed that the first 5, 10, and 20 components explained 55.6%, 74.8%, and 91.3% of the total variance, respectively, and loadings of the first 5 PCs are summarized in **Supplementary Fig. 4**.

### Model Performance

For the model predicting future (3-month) depressive symptoms as measured by the CESD, the best performing model based on RMSE and R2 was the multivariable linear regression model with the first 5 PCs as features (RMSE = 6.501, R2 = 0.688, Table 2 and Fig. 3). It achieved better performance compared with the univariable model using baseline CESD as the only predictor (RMSE = 6.750, R2 = 0.645). Across all models using *raw* features as input, the random forest model and ensemble approach had the best prediction accuracy, achieving R2 of 0.614 and 0.616, and RMSE of 6.723 and 6.738, respectively.

For the MFQ model, the best performing model based on RMSE and R2 was the simplest model: the univariable linear regression model with baseline MFQ total scores as the sole predictor (RMSE = 8.054, R2 = 0.671). The best multivariable model was also the linear regression model with the first 5 PCs as features (RMSE = 8.128, R2 = 0.647), but the performance was worse than the univariable model. Using raw features as input, the random forest model achieved the best prediction accuracy, with R2 of 0.562 and RMSE of 8.616.

### Feature Importance

**Figure 4:**
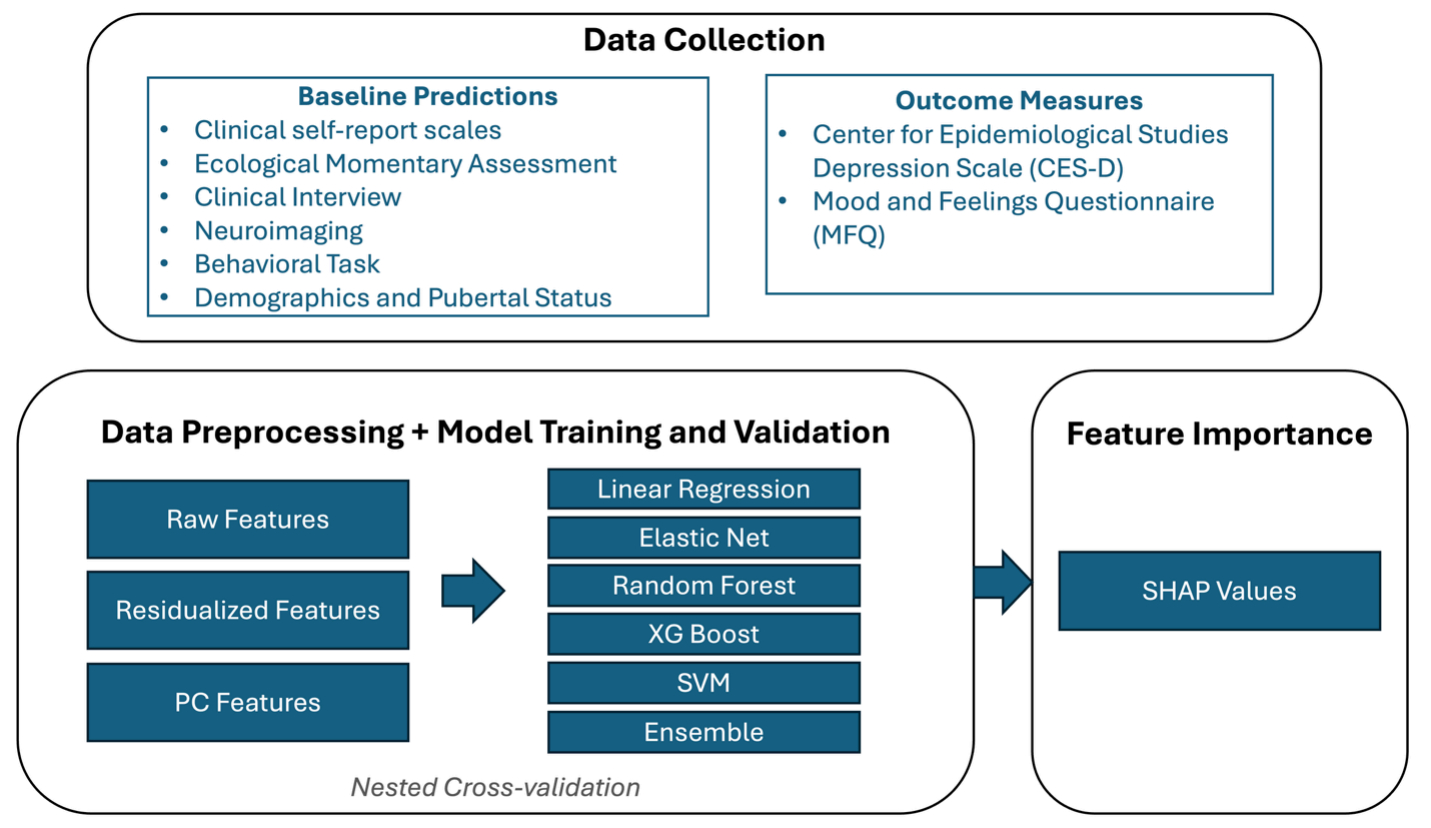
SHAP value-based feature importance for prediction of depressive symptoms at 3 months follow-up based on Random Forest model trained onraw features, with (a) CESD or (b) MFQ as the outcome measure. (Left) The barplot summarizes the overall impact of each feature on model output, ranked from the highest. (Right) The individual dot color corresponds to the value of the variable, and location on the plot’s *x* axis corresponds to that point’s relative impact on the model output. A high-feature value (red) with a corresponding high *x* axis value (SHAP value) represents a point that strongly, positively influences the model’s prediction of depression outcome.

To evaluate feature importance of each predictor variable, we fit a random forest model using all raw features on the full dataset (i.e. outside of the nested cross-validation framework). We focused on the random forest model since that raw feature model performed the best across the two depression outcome measures. For the model predicting CESD scores at 3 months, depressive (MFQ) symptom severity at baseline appears as the most influential feature, with a mean SHAP value five times greater than the second most influential feature, the baseline CESD score (Fig. 4a). Other features such as greater physical anxiety (MASC), anhedonia (SHAPS), and blunted striatal (right caudate) response to the anticipation of rewards also demonstrated predictive influence.

For the model predicting MFQ scores at 3 months, greater baseline depressive (MFQ) symptoms again emerged as the most important contributor to the predicted outcomes (Fig. 4b). The set of top influential features shows some overlap with those important for predicting MFQ scores, including greater physical anxiety (MASC), neuroticism (NEO), and blunted striatal (right caudate and putamen) response to the anticipation of rewards.

### Depression Symptom Factors

Given that baseline depression was the most robust predictor of future depression we sought to understand if there were particular factors or subsets of depression symptoms/items that were most predictive. For the CESD questionnaire, the first factor described depressed mood. The second factor was related to depressogenic social cognitions (e.g., “*I felt that people disliked me*”). The third factor described anhedonia, while the last factor was related to sleep issues and fear (**Supplementary Fig. 6a**). Within the MFQ questionnaire, the first factor reflected items related to melancholia, characterized by psychomotor changes (restlessness and psychomotor retardation), sleep difficulties, and difficulty concentrating. The second factor was related to low self-esteem and self-deprecation. The third factor points to items related to suicidal ideation, while the fourth factor included anhedonia symptoms and hopelessness (**Supplementary Fig. 6b**).

We explored whether certain factors were more strongly related to future depression. For the CESD model, only Factor 2 scores (depressogenic social cognitions) significantly correlated with the total CESD scores at 3 months (r^2^ = 0.16, p = 0.0014, **Supplementary Fig. 6a**). In the MFQ model, only factor 1 (melancholia) baseline scores significantly correlated with total MFQ scores at three months (r^2^ = 0.33, p = 1.3e-6, **Supplementary Fig. 6b**).

## Discussion

Early identification of depression risk is crucial for the timely implementation of prevention strategies, particularly during adolescence, a developmental period marked by heightened vulnerability to depressive symptoms. A key challenge in predicting future depression is its complex, multidimensional nature. Depression is influenced by a wide array of factors spanning psychological, behavioral, biological, and environmental domains, making it important to integrate data from multiple modalities when developing predictive models of future symptoms.

In this study, we explored machine learning approaches to predict future depressive symptoms in youth by integrating a wide range of theoretically-relevant predictors, including from conventional self-report scales (e.g., neuroticism and anhedonia), ecological momentary assessments (EMA; e.g., mean and variance in negative and positive affect), behavioral tasks (e.g., reward responsiveness), neuroimaging data (e.g., reward circuitry response), demographic characteristics (e.g., age and sex), and developmental measures (e.g., puberty status). Additionally, we employed machine learning (ML) techniques, combined with dimensionality reduction of baseline predictors, to enhance model accuracy and interpretability. In an effort to further improve predictive performance, this approach not only integrated numerous predictors, but also captured potential non-linear effects and interactions that studies using fewer predictors might overlook. Our use of a multivariable approach, combined with an analysis of feature importance, enabled a detailed assessment of which predictors exerted the most significant influence on depression outcomes.

For the CES-D, the multivariable linear regression model that used the first five principal components as predictors achieved the best performance, with an RMSE of 6.501 and an R^2^ of 0.688. This model outperformed the univariable approach, which only included baseline CES-D scores as the sole predictor. Previous research on predicting depression outcomes in treatment-seeking populations has similarly shown that multivariable models outperform univariable ones [50]. Our findings contribute to this body of evidence by extending these results to an adolescent sample recruited from the community, using a wide range of predictors.

In contrast, for the MFQ, the best-performing model was a simple univariable linear regression that used baseline MFQ scores as the sole predictor, achieving an RMSE of 8.054 and an R^2^ of 0.671. In line with this, feature importance analysis in the multivariable models indicated that baseline depressive symptoms, as measured by the MFQ, was the most robust predictor of future depressive symptoms. This finding aligns with previous research [17] and highlights the critical role that the severity of initial symptoms plays in predicting future depression outcomes (at least within the relatively short timeframe of 3 months). This emphasizes the importance of early symptom assessment as a key factor in forecasting long-term mental health trajectories.

Interestingly, feature importance showed that baseline MFQ was the best predictor of both CES-D and MFQ outcomes, even exceeding the predictive value of baseline CES-D for future CES-D scores. One explanation is that the MFQ is specifically designed for adolescents and covers a broader range of symptoms highly relevant to this age group. In addition, because the MFQ assesses symptoms over a two-week period rather than one week, it may provide a more stable index of severity. Together, these features may capture more trait-like aspects of adolescent depression vulnerability, whereas the CES-D may be somewhat more sensitive to short-term, state-related changes in symptoms.

While the univariable model using baseline depressive symptoms as the sole predictor performed best for MFQ, a random forest model incorporating all raw features also showed relatively strong performance in predicting both CES-D and MFQ depression scores. Feature importance revealed that the variables contributing most strongly to depression predictions were from conventional self-report measures (e.g., baseline depression severity, neuroticism and physical anxiety), EMA (mean positive affect) and neuroimaging (striatal response to the anticipation of rewards). These findings reinforce the multifaceted nature of depression, where a combination of psychological, biological, and behavioral factors contribute to its trajectory.

The observed importance of physical anxiety and neuroticism emphasizes the need to incorporate psychological characteristics into predictive models, which are relatively easily assessed via low-cost self-report measures, supporting the idea that depression risk is influenced by a constellation of internal and external factors [16]. These results align with previous research showing that depression is prospectively predicted by psychological variables, particularly neuroticism [51]. Moreover, findings also align with recent work suggesting that a blunted striatal response to reward is a hallmark of maladaptive reward processing in major depressive disorder [52], which may precede and predict future depression in youth [53]. However, when comparing across modalities, the bulk of the top predictors were self-report measures (CESD: 6/10 conventional self-report questionnaires and 1/10 EMA; MFQ: 6/10 conventional self-report questionnaires and 2/10 EMA). The relatively poor predictive performance of neural and behavioral measures may be due at least in part to fundamental differences in measurement reliability and error characteristics across modalities, as self-report measures have been shown to have higher reliability than single-session behavioral [54–57] or neuroimaging tasks [58]. Lower reliability in these tasks is in part due to low between-person variance, which can attenuate predictor-outcome associations. This reflects a fundamental design feature of experimental tasks, which are optimized to detect robust group-level condition effects at the expense of minimizing individual differences (which ultimately hampers reliability). Also, laboratory-based neural and behavioral measures may not capture real-world functioning as effectively as self-reported symptoms and traits. These findings highlight both the robust predictive value of carefully selected self-report measures and the need for improved approaches to capturing meaningful individual differences in neural and behavioral functioning.

Given the robust role of baseline depressive symptom severity in predicting future symptoms, with the univariable model performing the best for predicting MFQ scores, and baseline depression being the strongest predictor of CES-D scores in that multivariable model, we wanted to more closely examine which elements of depression were driving these effects. Therefore, we conducted a factor analysis of baseline MFQ and CES-D scores. Our analysis identified several distinct dimensions of depressive symptoms, including melancholia, low self-esteem, suicidal ideation, and anhedonia. These factors resemble those uncovered in previous research using the MFQ, namely vegetative symptoms, suicidality, cognitive symptoms, and agitated distress [59, 60]. The strong correlation of the melancholia MFQ factor with future MFQ total scores, and the depressogenic social cognition CES-D factor with future CES-D total scores, suggest that it may be important to emphasize targeting these specific symptom dimensions in preventive interventions.

Our results showed that complex ML approaches did not outperform regression-based techniques, aligning with prior work [50]. However, tree-based machine learning models such as random forest performed relatively well and provided complementary insights via SHAP-based feature importance analyses. These models may still offer meaningful value, particularly in larger samples or when interactions and nonlinear relationships are more prominent. More research is needed to determine in which contexts ML approaches are likely to outperform simpler statistical models (e.g., see [61, 62] for examples of ML models incorporating passive smartphone sensor data which outperformed simpler statistical approaches in predicting negative affective states). Future studies could benefit from assessing other depression-relevant baseline individual characteristics which were not assessed in this study that may improve predictive performance (e.g., emotion regulation abilities, sleep habits, home and school environment, early adversity, or executive function). Furthermore, there are of course factors in the intervening period *between* baseline and follow-up assessments that were not captured in our study which are known to be robust predictors of depression (e.g., stressors and negative life events, especially in the interpersonal domain).

The current study had several limitations. One notable limitation of our study is the relatively small sample size, in particular for the multivariable ML models. Although this risk was somewhat mitigated through dimensionality reduction of baseline predictors and the use of penalized ML models, a larger sample size is needed to more reliably estimate these models [63, 64]. Relatedly, while we used a nested cross-validation approach to mitigate overfitting, external validation in a larger, independent sample will be important to further evaluate the generalizability of these findings. Second, although our current sample is relatively diverse (38% non-White), it may not fully represent the broader adolescent population, which could limit generalizability. Another limitation is the short follow-up period, with only a single follow-up assessment after three months. This design restricts our ability to capture longer-term depression symptoms [65] and assess the stability of the predictive factors over time. Additionally, our study used two different depression outcome measures, CES-D and MFQ, analyzing them separately. This approach may complicate clinical interpretation and the integration of findings. Future research should explore methods for combining or integrating different outcome measures (including clinical interviews) to enhance the clinical applicability of predictive models and provide a clearer understanding of depression dynamics.

These limitations notwithstanding, the present study demonstrated the utility of models that integrate diverse predictors from psychological, behavioral, developmental, and neural assessments to characterize risk for future depression and their relative predictive importance. Further research is needed to test the generalizability of these models across multiple time scales and populations.

## Supplementary Material

Supplementary Files

This is a list of supplementary files associated with this preprint. Click to download.

- WangsupplementR1clean.docx

## Figures and Tables

**Table 1:** Demographic and Clinical Characteristics of the Sample

Sample Characteristics		
	N	%
**Biological Sex**		
Female	75	72.8
Male	28	27.2
**Race**		
American Indian or Alaska Native	0	0.0
Asian	13	12.6
Black or African American	11	10.7
Native Hawaiian or Other Pacific Islander	1	1.0
White	64	62.1
Other	3	2.9
More than one race	10	9.7
Unknown race	1	1.0
**Ethnicity**		
Hispanic or Latino	10	9.7
Not Hispanic or Latino	92	89.3
Unknown Ethnicity	1	1.0
**Current Diagnoses (DSM-V)**		
Major Depressive Episode	0	0.0
Generalized Anxiety Disorder	5	4.9
Social Anxiety Disorder	3	2.9
Panic Disorder	1	1.0
Specific Phobia	0	0.0
Attention-Deficit / Hyperactivity Disorder	3	2.9
Obsessive Compulsive Disorder	2	1.9
**Medication**		
**Biological Sex**		
SSRI	2	1.9
	M	SD
**Age (in years)**	16.0	1.9
**Family Income (dollars)**	165,844.5	92,242.4
**Baseline SHAPS Score**	23.0	7.4
**Baseline CES-D Score**	12.3	11.1

*Note*. The SHAPS is scored on a 1–4 scale where higher scores indicate greater anhedonia; the possible range is 14–56. The CES-D is scored on a 0–3 scale where higher scores indicate greater depression; the possible range is 0–60.

**Table 2:** Model performances for prediction of depressive symptoms (CESD and MFQ) at 3 months follow-up.

Data type	Model	CESD			MFQ		
		R2	RMSE	MAE	R2	RMSE	MAE
Univariable	Linear Regression	0.645	6.750	4.908	**0.671**	**8.054**	5.769
Multivariable- Raw	Linear Regression	0.502	8.589	6.631	0.469	10.812	8.210
	Elastic Net	0.570	7.098	5.217	0.521	9.019	6.002
	Random Forest	0.614	6.723	4.808	0.562	8.616	5.764
	XGBoost	0.545	7.476	5.209	0.543	9.039	5.966
	SVM	0.510	8.134	5.937	0.459	9.985	7.101
	Ensemble	0.616	6.738	4.819	0.549	8.755	5.697
Multivariable-Residuals	Linear Regression	0.323	8.891	6.574	0.268	11.11	7.941
	Elastic Net	0.540	7.327	5.451	0.524	8.969	6.117
	Random Forest	0.503	7.621	5.447	0.558	8.635	5.893
	XGBoost	0.332	8.833	6.359	0.464	9.510	6.459
	SVM	0.396	8.395	6.215	0.378	10.249	7.331
	Ensemble	0.544	7.301	5.302	0.541	8.805	5.849
Multivariable-PC 5	Linear Regression	**0.688**	**6.501**	4.873	0.647	8.128	5.931
	Elastic Net	0.591	6.912	4.919	0.511	9.093	5.937
	Random Forest	0.606	6.785	4.932	0.535	8.866	5.961
	XGBoost	0.463	7.891	5.651	0.343	10.523	6.753
	SVM	0.602	6.823	4.806	0.509	9.106	5.817
	Ensemble	0.600	6.836	4.846	0.528	8.933	5.931
Multivariable-PC 10	Linear Regression	0.588	7.204	4.823	0.521	9.356	5.812
	Elastic Net	0.584	6.976	5.014	0.534	8.869	5.792
	Random Forest	0.604	6.807	5.022	0.541	8.801	5.883
	XGBoost	0.453	7.982	5.672	0.426	9.792	6.404
	SVM	0.616	6.705	4.708	0.557	8.649	**5.450**
	Ensemble	0.623	6.638	**4.685**	0.547	8.752	5.683
Multivariable-PC 20	Linear Regression	0.546	7.468	5.078	0.477	9.248	6.289
	Elastic Net	0.562	7.156	5.312	0.529	8.922	5.811
	Random Forest	0.580	7.004	5.254	0.506	9.132	6.234
	XGBoost	0.425	8.190	5.911	0.364	10.341	6.779
	SVM	0.589	6.929	5.007	0.550	8.722	5.662
	Ensemble	0.575	7.046	5.096	0.522	8.982	5.959

Results are summarized over 30 repetitions. RMSE: root mean squared error, R2: R squared, MAE: mean absolute error. Univariable: model with respective baseline depression as the sole predictor, Raw: all normalized baseline variables as predictors, Residuals: residualized baseline variables as predictors, PC5: first 5 principal component scores, PC10: first 10 principal component scores, PC20: first 20 principal component scores. The best performance (highest R2, lowest RMSE and MAE) across all data pre-processing procedures are bolded, and the best performance with “Raw” features are underlined.

## Data Availability

Mass General Brigham requires IRB approval and a signed Data Use Agreement for data sharing. Please contact the first author.
